# The surgical strategy for eosinophilic granuloma of the pediatric cervical spine complicated with neurologic deficit and/or spinal instability

**DOI:** 10.1186/s12957-016-1063-6

**Published:** 2016-12-07

**Authors:** Nanzhe Zhong, Wei Xu, Tong Meng, Xinghai Yang, Wangjun Yan, Jianru Xiao

**Affiliations:** Department of Orthopedic Oncology, Changzheng Hospital, Second Military Medical University, 415 Fengyang Road, Shanghai, 200003 China

**Keywords:** Pediatric, Cervical, Eosinophilic granuloma, Surgery

## Abstract

**Background:**

Various therapeutic approaches have been proposed for the treatment of pediatric patients with eosinophilic granuloma (EG) of the cervical spine. Our aim was to discuss and present our experience with the individualized surgical intervention of pediatric cervical EG complicated with neurologic deficits and/or spinal instability.

**Methods:**

We retrospectively analyzed the clinical data of 19 children who were diagnosed with cervical EG comor spinal/or spinal instability (evaluated by the Spinal Instability Neoplastic Score, SINS ≥ 7) and treated surgically in our institution.

**Results:**

Lesions involved C1–2 in 7 patients and C3–7 in 12 patients. Anterior tumor resection combined with posterior pedicle screw fixation, anterior approach of excision and instrumentation, and posterior tumor resection combined with pedicle screws instrumentation were selected according to the different locations of tumors. Frankel scale and Oucher scale improved significantly after surgery. There was no morphologic alteration of the neck at follow-up.

**Conclusions:**

Surgery can significantly improve the neurologic status and symptoms. Surgical decision-making must be individually tailored to minimize the influence of surgery on spine growth.

## Background

Langerhans-cell histiocytosis (LCH) is a complicated disease entity that can result in either benign or devastating outcomes. Among the clinical spectrum of LCH, eosinophilic granuloma (EG) is the most benign and predominant condition, featured by a clonal proliferation of Langerhans-type cells with bone involvement. In children and young adolescents with this tumor, about 80% of patients are reported to be younger than 20 years [1].

Although thoracic segments is the most preferential spine localization in EG patients of all ages with vertebral involvement [1, 2], data of previous studies and our research has suggested that incidence in the cervical spine is the highest in pediatric patients with spinal EG [3, 4].

The typical vertebral lesion of EG is composed of an osteolytic area causing complete or incomplete collapse and flattening of vertebrae, known as vertebra plana. The bony destruction of vertebral EG might lead to spinal instability with severe mechanical back pain and even a pathological fracture. Pediatric patients with cervical EG can present with symptoms including acute onset of neck pain with/without history of trauma and torticollis or neck stiffness due to restricted motion [5, 6]. Vertebral collapse and impingement from extradural extension of the lesion may further result in nerve compression and subsequent neural deficit and myelopathy.

Various therapeutic approaches have been proposed for the treatment of pediatric patients with EG of the cervical spine. For most cases without neural deficits or spinal instability, it is often sufficient to palliate symptoms and induce the lesion regression by conservative treatment and biopsy with cervical collar immobilization in view of the possibility of spontaneous resolution. Surgical intervention should be considered to relieve pain, maintain spinal stability, and preserve neural function in the presence of neurologic compromise, spinal instability, and severe deformity [1]. It has been documented that several surgical strategies are feasible for the achievement of the abovementioned therapeutic goals in the treatment of pediatric cervical EG without growth arrest and ensuing deformity [5, 7]. However, as no randomized clinical trials have been performed on the pediatric cervical EG due to the scarcity of cases and ethical issues, there is no consensus about the optimal surgical procedure regarding surgical approaches and internal fixation methods.

**Fig. 1:**
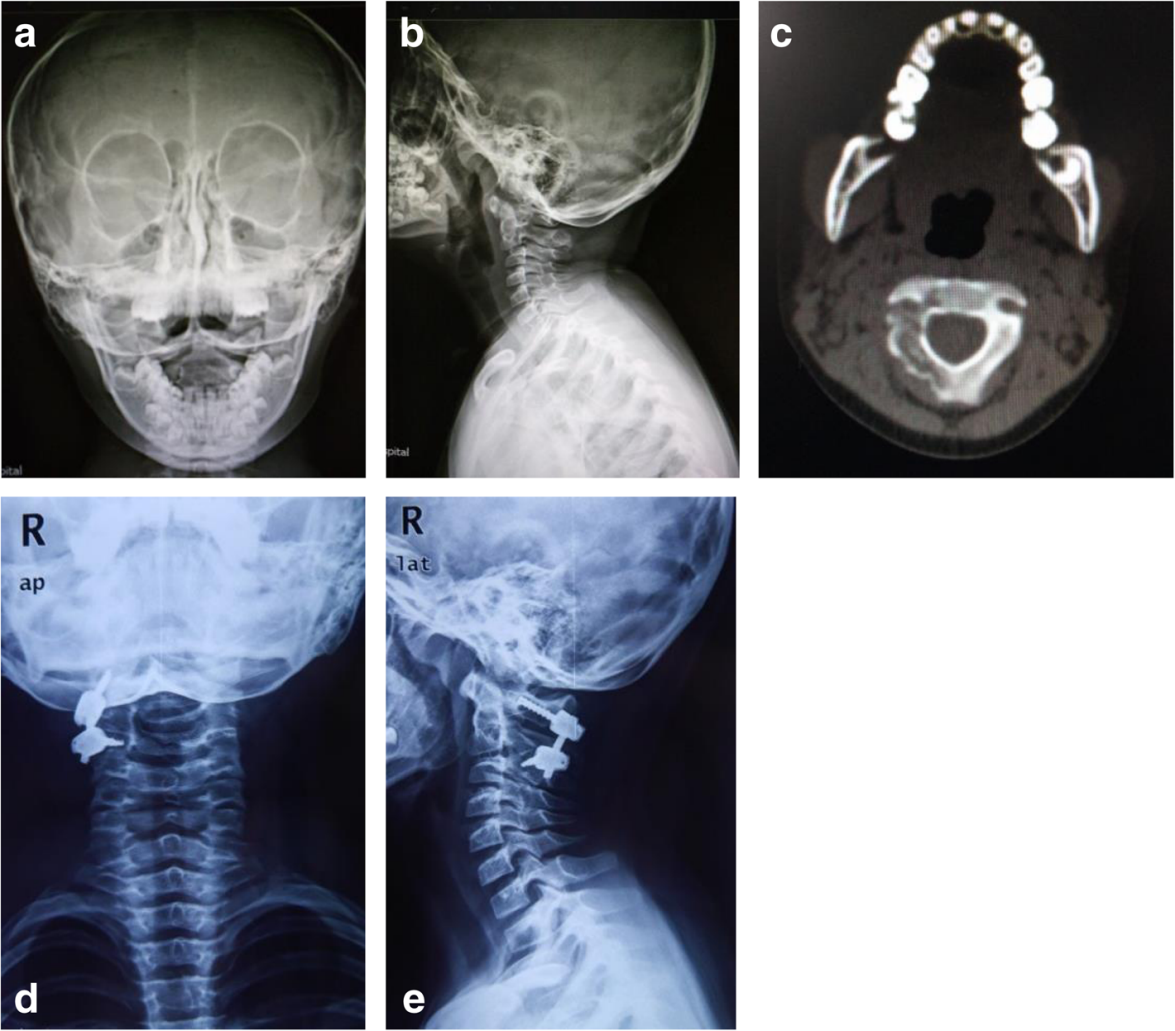
Case 10: a 5-year-old girl, C2 LCH. **a**, **b** Anterioposterior and lateral plain film before operation. **c** CT scan showed the right appendix of C2 was involved. **d**, **e** Anterioposterior and lateral plain film 48 months after operation of anterior tumor resection combined with posterior pedicle screw instrumentation

**Fig. 2:**
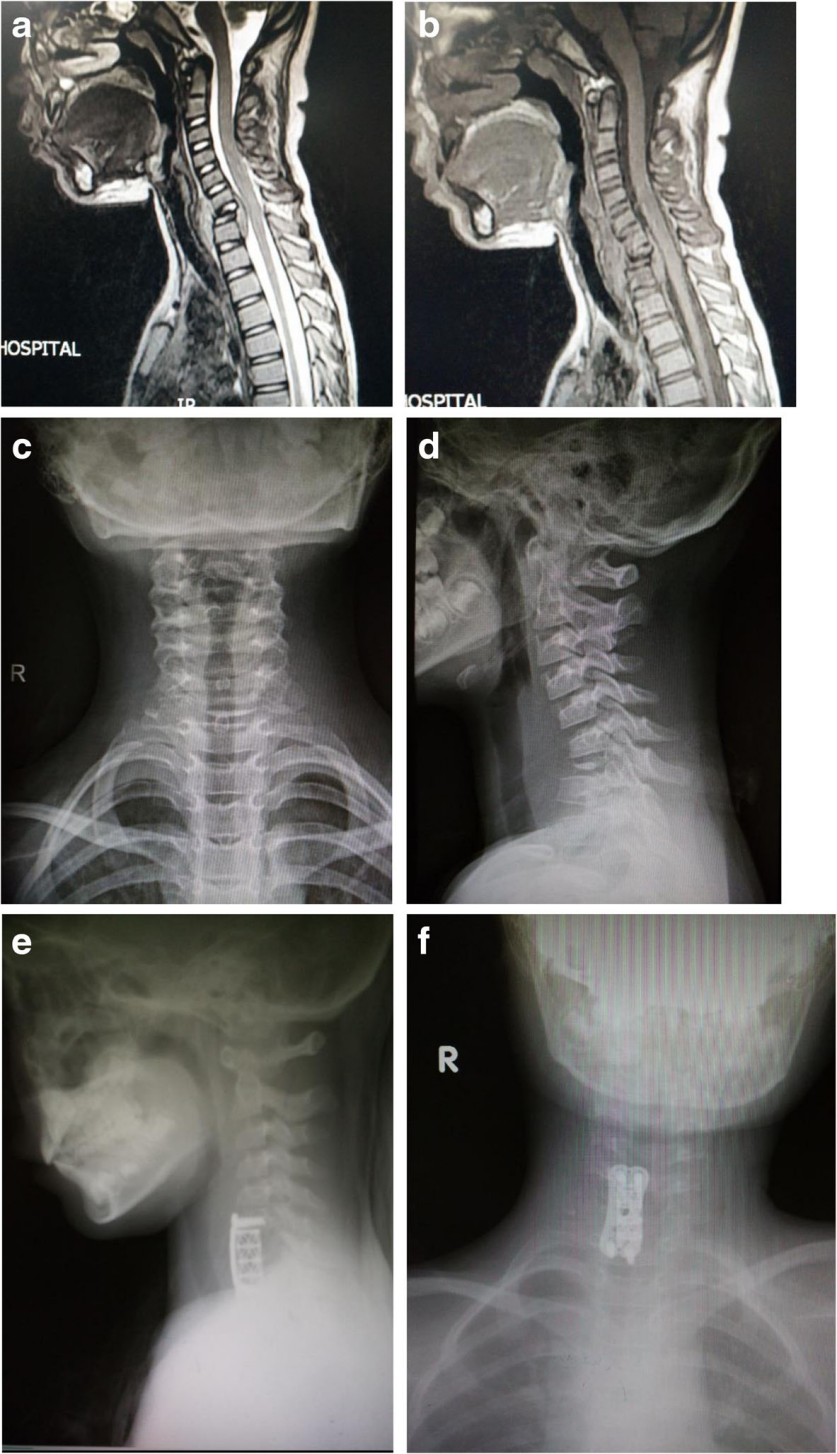
Case 4: a 7-year-old girl, C7 LCH. **a**, **b** T1- and T2-weighted MRI images before operation. **c**, **d** Anterioposterior and lateral plain film before operation. **e**, **f** Anterioposterior and lateral plain film 15 months after operation of anterior tumor resection and miniplate and screw instrumentation

To discuss the most suitable surgical management of pediatric patients with cervical EG, a retrospective study was undertaken reviewing 19 cases of cervical EG complicated with neurologic deficit and/or spinal instability. Herein, we reviewed the clinical features of pediatric cervical EG and presented our experience with the surgical intervention of the disease (Figs. 1 and 2).

## Methods

### Patient samples

This study was approved by the ethics committee of the Changzheng Hospital of Second Military Medical University. We retrospectively reviewed cases in 19 children (≤15 years) who were diagnosed with cervical EG complicated with neurological deficits and/or spinal instability (evaluated by the Spinal Instability Neoplastic Score, SINS ≥7 [8]) and treated surgically between April 2009 and June 2014 in our institution. The exclusion criteria were multiple systems of involvement and those arising in the thoracic and lumbar spine. A frozen biopsy is performed in every surgery to verify the diagnosis on the basis of preoperative clinical data. In addition to the frozen biopsy, a routine histopathologic biopsy is also performed to provide the definite diagnosis. Pain and neurological function were evaluated by Oucher scale and Frankel scale, respectively.

### Treatment (operative procedures)

The surgical approach was tailored for each patients depending on Weinstein-Boriani-Biagini (WBB) surgical staging system, including posterior approach, anterior approach, or a combination. The instrumentation method (anterior miniplate and screws or posterior pedicle screws) was also individualized in consideration of minimizing motion scarification and influence on growth. All surgeries were performed in one stage.

Postoperatively, patients were prescribed with prednisone 40 mg/m^2^/day orally days 1–5 every 3 weeks for a total treatment duration of 6 months. To reinforce the stability of the cervical spine, patients were immobilized with neck collars for at least 6 months until the fusion of the bones.

### Follow-up

All patients were followed up in the outpatient clinics at 3-, 6-, and 12-month postoperatively and annually for life thereafter. Outcome including radiographic image (anteroposterior and lateral cervical plain film and MRI) was investigated at each visit, and pubertal status (height, weight, bone maturity) was evaluated every 6 months. The neurologic status and pain were also assessed by Frankel scale and Oucher scale, respectively.

### Statistical analysis

All data were analyzed using SPSS 18.0 analysis software. To compare the pre- and postoperative clinical parameters and outcome, the paired-sample *t* test was used. Statistical differences are presented at when *p* values were <0.05.

## Results

The cohort consists of 15 boys and 4 girls. The average age of diagnosis was 9.9 ± 3.1 years (range, 5–15 years) with mean height and weight being 143.0 ± 21.2 cm and 36.8 ± 13.3 kg, respectively. The basic clinical data were summarized in Table 1. The mean time from onset to admission was 67.4 days. The results of preoperative laboratory tests showed elevation of erythrocyte sedimentation rate (ESR) in seven cases ranging from 5 to 44 mm/h (normal range, 0~15 mm/h) and elevation of C-reactive protein in six cases ranging from 0.97 to 42.77 mg/L (normal range, <1.0 g/L).

**Table 1:** Clinical data before treatment

No.	Gender	Age	Involved vetebrae	SINS	Frankel	Oucher	WBB	Height (cm)	Weight (kg)
1	M	11	C7	7	D	9	2	150	43
2	M	10	C7	13	D	7	1	145	38
3	M	6	C6	8	D	4	2	120	26
4	F	7	C7	14	D	6	1	110	18
5	M	6	C5	15	D	8	2	100	12
6	M	11	C2	16	D	8	1	152	45
7	M	9	C2	9	E	7	1	141	32
8	F	7	C2	16	C	10	1	122	20.5
9	M	15	C3	10	E	7	1	167	58
10	F	5	C2	12	E	8	1	120	26
11	M	14	C2	14	D	9	1	160	48
12	M	14	C2	14	D	8	1	163	47
13	M	13	C5	13	D	9	1	159	42
14	M	13	C4	12	C	8	1	168	53
15	M	11	C7	9	D	9	1	152	45
16	M	12	C4	9	D	10	1	169	54
17	M	10	C2	14	D	10	1	148	36
18	M	9	C4	10	C	8	2	151	34
19	F	6	C6	9	D	9	1	120	21

WBB: zones 4–9: 1, zones 10–5: 2

The initial clinical presentation was retrieved from the medical records. The most common initial symptoms were, in descending order, restricted cervical motion (100%), neck pain (94.7%), neurological symptoms (84.2%), and torticollis (26.3%). The average SINS score was 11.8 ± 2.5. Oucher scale for pain was obtained, with the mean score being 8.1 ± 1.5. The neurological status of all the patients was accessed and classified according to Frankel scale. The result reveals 3 patients with Frankel grade C, 14 with grade D, and 2 with grade E (Table 1).

### Radiological evaluation

Preoperative radiographic data of all patients were collected, including antero-posterior and lateral cervical roentgenograms, computed tomography (CT), and magnetic resonance image (MRI). In 7 patients, lesions involved the upper cervical spine (C1–2) and in 12 patients, lesions involved the lower cervical spine (C3–7). The distribution of the tumors was evaluated using the Weinstein-Boriani-Biagini (WBB) classification on the basis of layers and zones on CT and MRI findings. In most cases, the tumors involved layers A–D, with only six lesions extended to layers B–D. It is found that tumors were located majorly in the anterior column (zones 4–9) in 15 cases and the posterior column (zones 10–5) in 4 cases.

### Treatment and follow-up

The customized surgical strategy was designed for each patients depending on the location and extension of the lesion. For the atlantoaxial tumor, anterior tumor resection combined with posterior pedicle screws fixation was performed (seven cases). We selected anterior approach of excision and instrumentation for tumors majorly located on the anterior column (eight cases), and posterior tumor resection combined with pedicle screws instrumentation for those involved posterior elements (four cases).

The mean operation time was 217.1 ± 40.7, 169.8 ± 37.1, and 131.3 ± 35.7 min with the surgical procedure for atlantoaxial LCH, anterior tumor removal and miniplate and screw instrumentation, and posterior tumor resection and pedicle screw fixation, respectively. The mean intraoperative blood loss was 241.4 ± 191.0 mL with the combined procedure for atlantoaxial LCH, 165.0 ± 69.5 mL with single anterior approach, and 137.5 ± 45 mL with single posterior approach. There was no intraoperative complication noted in all patients. Of all patients in our series, no instrumentation failure and cervical deformity was seen at a mean follow-up time of 36.4 ± 13.7 months (range 13–53 months), with a median follow-up time of 41 months. The average height and weight at the last follow-up were 158.1 ± 14.9 cm and 50.3 ± 14.4 kg, respectively (Table 2). Unfortunately, recurrence on thoracic vertebrae was detected 6 months after surgery in one child who did not take prednisone due to remission of symptoms and being afraid of the adverse effects of steroid. She was cured with oral prednisone and brace. All patients with neurological deficits got complete recovery from 1 to 2 weeks after surgery without deterioration during follow-up, illustrated by the significantly improved Frankel grade (*p* < 0.001). Pain was significantly released by treatment, with average Oucher score decreasing from 8.1 ± 1.5 to 0.3 ± 1.4 (*p* < 0.001) (Table 2). The average duration of the cervical collar immobilization was 4.4 months. Three boys stopped wearing the cervical collar once they had resolution of symptoms. However, bone fusion was achieved in all cases without instrumentation failure. There was no morphologic alteration of the neck and no limitation in rotational movement of the cervical spine observed at follow-up.

**Table 2:** Clinical data of operation and at the last follow-up

No.	Surgical procedure	Operative time (min)	Blood loss	Follow-up (month)	Height (cm)	Weight (kg)	Frankel	Oucher	Adjuvant therapy	Recurrence
1	3	135	160	53	173	63	E	0	Oral pred	No
2	2	179	100	18	152	46	E	0	Oral pred	No
3	3	160	100	26	135	37	E	0	Oral pred	No
4	2	180	160	15	132	26	E	0	Oral pred	No
5	3	80	100	38	136	33	E	6	–	Yes
6	1	240	200	52	175	60	E	0	Oral pred	No
7	1	240	400	49	156	49	E	0	Oral pred	No
8	1	210	100	47	151	38	E	0	Oral pred	No
9	2	170	300	13	170	64	E	0	Oral pred	No
10	1	130	80	48	141	33	E	0	Oral pred	No
11	1	250	600	43	172	64	E	0	Oral pred	No
12	1	230	120	41	175	69	E	0	Oral pred	No
13	2	250	200	50	171	67	E	0	Oral pred	No
14	2	180	200	46	174	70	E	0	Oral pred	No
15	2	134	100	37	165	62	E	0	Oral pred	No
16	2	145	150	14	171	56	E	0	Oral pred	No
17	1	220	190	25	152	39	E	0	Oral pred	No
18	3	150	190	35	154	42	E	0	Oral pred	No
19	2	120	110	42	149	37	E	0	Oral pred	No

Surgical procedure: *1*: anterior tumor resection combined with posterior pedicle screw instrumentation; *2*: anterior tumor resection and miniplate and screw instrumentation; *3*: posterior tumor resection and pedicle screw instrumentation

## Discussion

LCH can occur in any age groups but is more common in the pediatric population, with the estimated incidence ranging from 2 to 10 per million children [9, 10]. Although EG of the cervical spine is a rare condition, there is a predominant involvement of cervical spine in pediatric patients with spinal EG [3, 4]. The typical pattern of vertebral destruction can facilitate the establishment of the diagnosis of cervical EG. Nevertheless, a tissue biopsy for histopathologic examination is required to diagnose EG formally. For patients with unifocal osseous lesions, the overall benefits of needle or open biopsy remains controversial [1]. In our practice, for pediatric cervical EG patients suffered from neurological deficits and/or had SINS ≥7, we suggested surgery as an emergent treatment choice to prevent these children from any possible impending disastrous damages. And a frozen biopsy was performed in these surgeries to further clarify the diagnosis on the basis of preoperative clinical data, so as to reconfirm the previously designed surgical modality, or to make a modification accordingly. In addition to the frozen biopsy, a postoperative routine histopathologic examination was also performed to provide a definite diagnosis.

Given the general benign prognosis of spinal EG [11], various therapeutic modalities were employed in pediatric patients without neurological deficits or spinal instability, including chemotherapies, low-dose radiotherapies, and minimally invasive treatments [12–16]. Although lack of consensus, it is generally considered that spine instability, kyphoscoliosis, and neurologic deficiencies and neurologic deficit are major surgical indications for spinal EG [1]. To avoid further disastrous damages resulted from spinal cord compression and cervical instability, we regarded neurologic deficiencies and SINS ≥7 as indications for treating pediatric cervical EG surgically.

Surgical modalities for cervical EG may vary with the extent and location of the disease. For EG of the atlas and axis in adult patients, we have previously reported that anterior tumor resection and posterior reconstruction by posterior occipitocervical fixation or upper cervical fixation can offer eradication of the lesion, decompression of nerve, and immediate atlantoaxial stability [17]. However, in view of preserving the potential growth of spine and improving quality of life, this procedure was performed for pediatric patients with upper cervical EG in our series. C1 and C2 pedicle screw instrumentation technique has been proved to be safe with low complications when it is applied on selected pediatric patients [18]. The data of our study reinforces the statement that this method of fixation can be a safe and effective alternative for pediatric upper cervical EG, minimizing the influence on spine growth and motion.

In comparison, more reports focused on the surgical procedures for lower cervical EG, which included curettage and fusion with or without instrumentation. Denaro et al. [5] described specially modeled autoplastic grafts harvested from the iliac wing, which were inserted after complete excision of the anterior portion of the EG involved vertebral body to maintain normal motion of the adjacent segments and growth of the vertebrae in the long term. Arthrodesis was achieved in patients after immobilization by Minerva cast for 90 days. To handle lesions with greater extent of vertebral destruction, anterior corpectomy would be performed followed by mesh cage and plate instrumentation to obtain fusion in both pediatric and adult patients [7, 19]. The nuance between this surgery performed in different age group is that the longest possible mesh cage is recommended in children considering spine growth and restoration of the cervical lordosis curve. The principle of our surgical consideration for pediatric cervical EG is minimizing the insults of surgery on the growing spine. For lesions located on the anterior column of the segment of the lower cervical spine, anterior resection of tumor was performed. To reconstruct the stability of spine, autologous bone graft or mesh cage was inserted on the basis of the extension of resection, and anterior mini-plate and screw were used. Correspondence to prior reports, there is no adverse effect on spine curvature detected during the follow-up and the remission of symptoms was quick [7, 20]. If only the posterior element was destroyed by the tumor, we recommend posterior excision and pedicle screw instrumentation, which is featured by short operation time and small amount of bleeding. It is suggested by Lu et al. that pedicle screw-based instrumentation and the independent posterior approach is a preferable procedure for children with thoracic and lumbar EG for its minimal trauma and favorable results [21]. Our data supported the conclusion of Lu’s study and further proves that posterior approach of tumor removal and instrumentation is also an ideal choice for the individual surgical treatment of selected pediatric cervical EG cases.

There has been no definitive evidence-based conclusion yet to guide the adjuvant therapy after surgical intervention of spinal EG. Opinions vary from only observation to low-dose standard chemotherapy. On the basis of the experience drawn from our clinic and previous report [13], desirable effects and high rate of adherence can be acquired by orally taking prednisolone alone. Furthermore, one patient in the current study who was not compliant with oral prednisolone had recurrent EG on the thoracic vertebrae, suggesting the necessity of systemic intervention of spinal EG instead of only local excision of the tumor. The duration of postoperative immobilization is also under investigation. Patients were asked to wear cervical collars for 6 months to ensure spinal fusion. However, no spinal deformity or instrumentation failure was observed even in some children who had worn orthosis for only 1 or 2 months.

## Conclusions

There is an obvious demand for a full assessment of each pediatric cervical EG patient with a rational surgical procedure tailored to the extension and location of the lesion. Limitations of the current study are common to retrospective research in which the data are only as precise as available medical records. Besides, our study has limited power as a result of small numbers and uncontrolled variables. Despite the limitations, our results strongly suggest the application of aforementioned procedures on the specific site of cervical spine and contribute to the individualized surgical treatment of pediatric cervical EG patients complicated with neurologic deficit and/or spinal instability, which is characterized by minimal surgical trauma and effects on spine growth without recurrence. The neural status and symptoms of these patients were significantly improved by the surgical intervention and EG-related cervical deformity was corrected by instrumentation. After all, surgeries for pediatric cervical spine is demanding and require thorough pre-operative planning and special technical expertise to supply an ensurance for the safety and effectiveness both in the operation and in the long term.

## Abbreviations

EG: Eosinophilic granuloma
ESR: Erythrocyte sedimentation rate
LCH: Langerhans cell histiocytosis
SINS: Spine Instability Neoplastic Score
WBB: Weinstein-Boriani-Biagini
